# Supplementary material for: Carbon and health implications of trade restrictions
Source: Nat Commun. 2019 Oct 30;10:4947. doi: 10.1038/s41467-019-12890-3 (PMC6821914; doi:10.1038/s41467-019-12890-3)
Supplement: Supplementary file 2 — Supplementary Information [file 41467_2019_12890_MOESM2_ESM.docx]

**Supplementary Information for**

**Carbon and health implications of trade restrictions**

Lin et al.

# Supplementary Figures

Supplementary Figure 1: Regional emission total (left axis) and emission intensity (right axis) for each species in global free trade scenario (GFT). Error bars denote uncertainty ranges (95% CI) of emissions. Results only include scenario-dependent sources. Regions include China (CH), rest of East Asia (EA), Economies in Transition (ET), Japan and Korea (JK), Latin America and Caribbean (LA), Middle East and North Africa (MN), rest of North America (NA), Oceania (OC), South Asia (SA), South-East Asia and Pacific (SE), Sub-Saharan Africa (SS), the United States (US), and Western Europe (WE).

Supplementary Figure 2: Regional emission intensities as a function of regional per capita GDP for CO_2_ and six pollutants in actual trade restriction scenario (ATR). Emissions here include scenario-dependent sources. Regions include China (CH), rest of East Asia (EA), Economies in Transition (ET), Japan and Korea (JK), Latin America and Caribbean (LA), Middle East and North Africa (MN), rest of North America (NA), Oceania (OC), South Asia (SA), South-East Asia and Pacific (SE), Sub-Saharan Africa (SS), the United States (US), and Western Europe (WE).


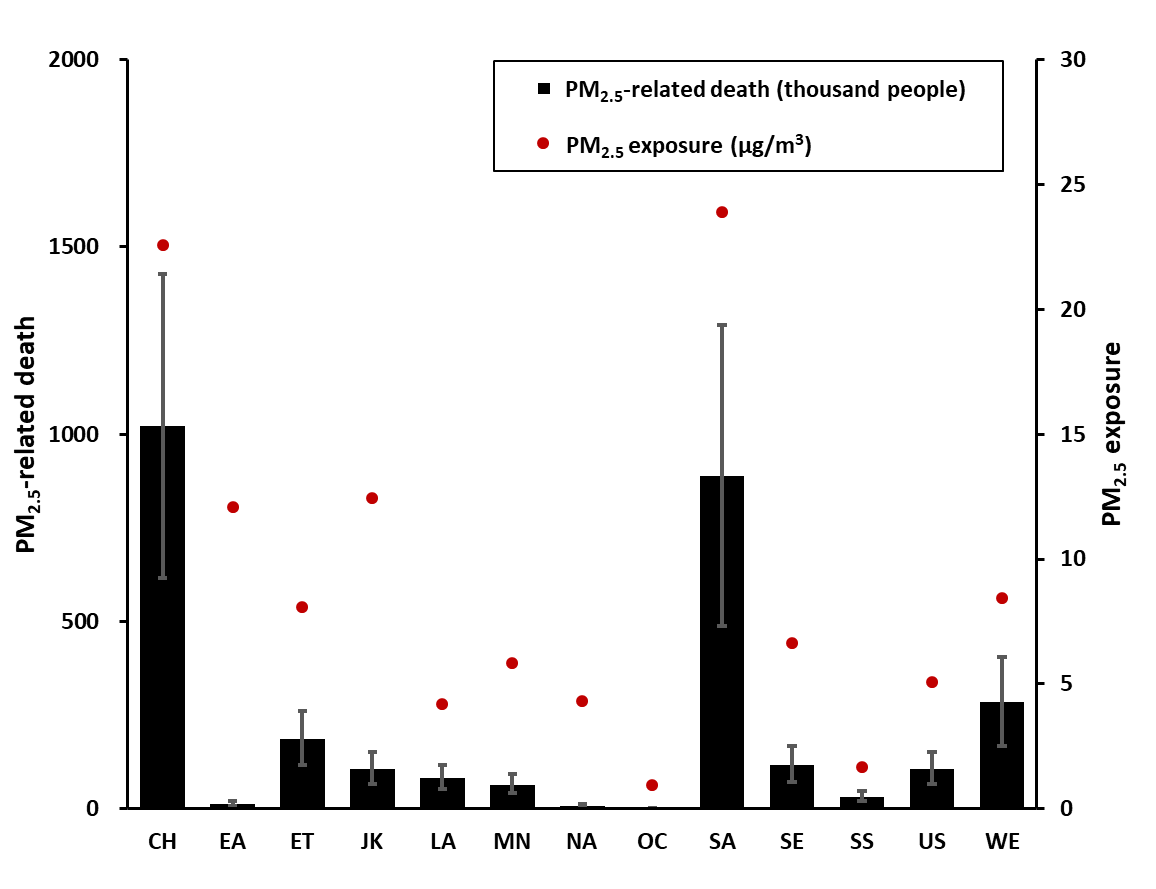


Supplementary Figure 3: Regional premature deaths (left axis) and PM_2.5_ concentrations (right axis) in global free trade scenario (GFT). Error bars denote uncertainty ranges (95% CI) of deaths. PM_2.5_ considered here only include scenario-dependent SIOA, BC and POA together. Regions include China (CH), rest of East Asia (EA), Economies in Transition (ET), Japan and Korea (JK), Latin America and Caribbean (LA), Middle East and North Africa (MN), rest of North America (NA), Oceania (OC), South Asia (SA), South-East Asia and Pacific (SE), Sub-Saharan Africa (SS), the United States (US), and Western Europe (WE).


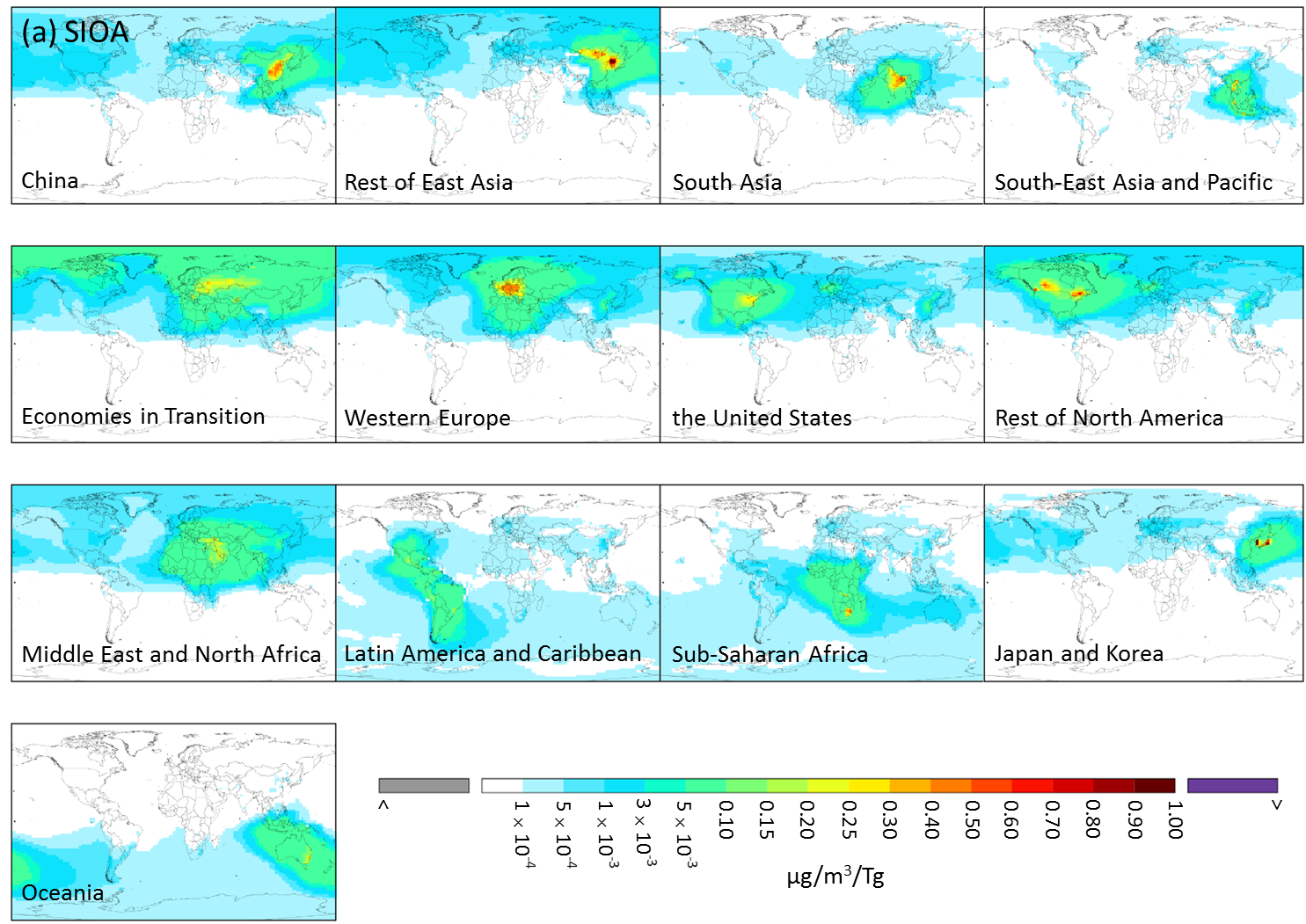

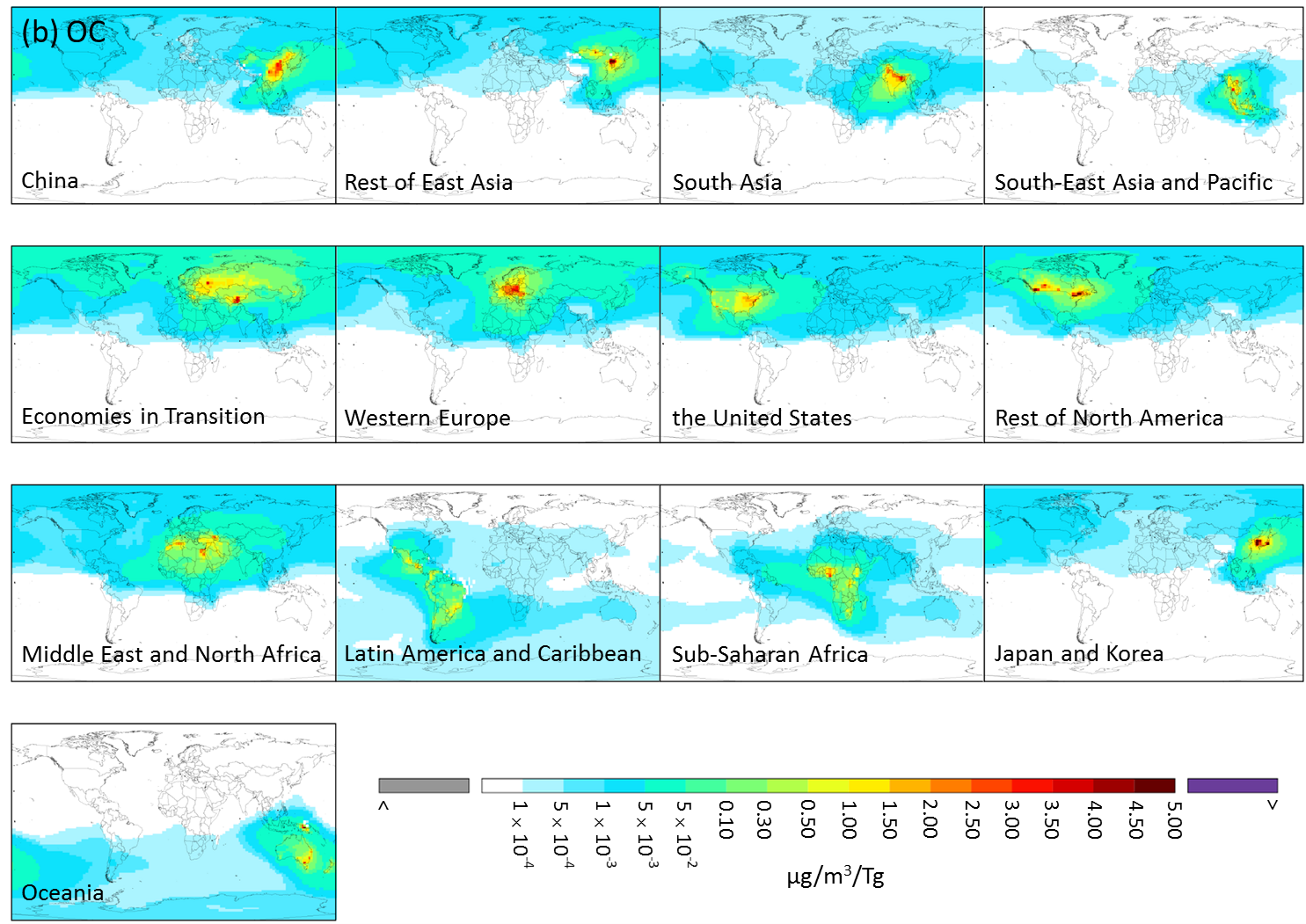


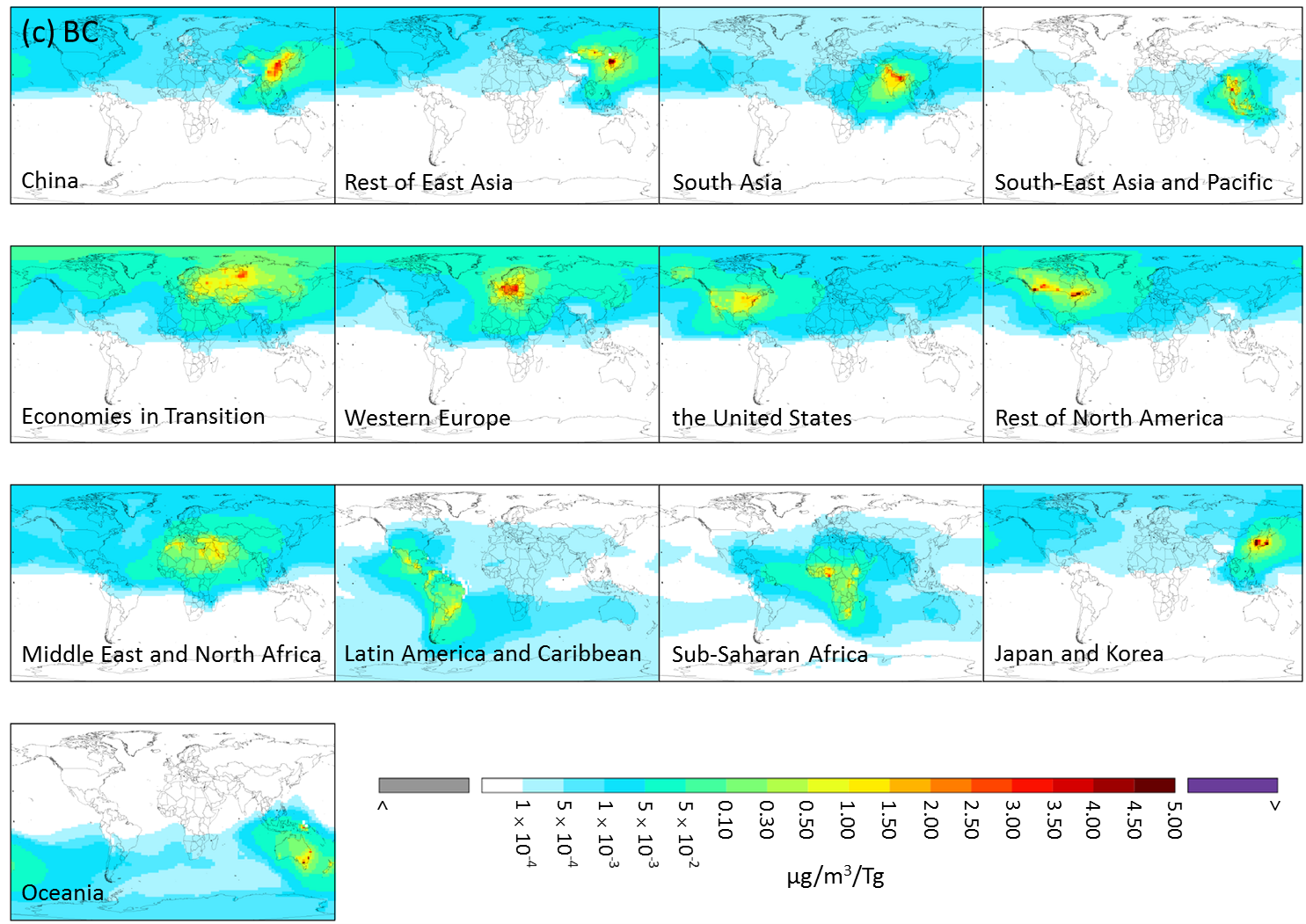


Supplementary Figure 4: Chemical efficiency (μg/m^3^/Tg) of scenario-dependent (a) SIOA, (b) OC and (c) BC, calculated as pollution worldwide caused by one unit of emissions released from each source region. Note that the color scales are nonlinear.


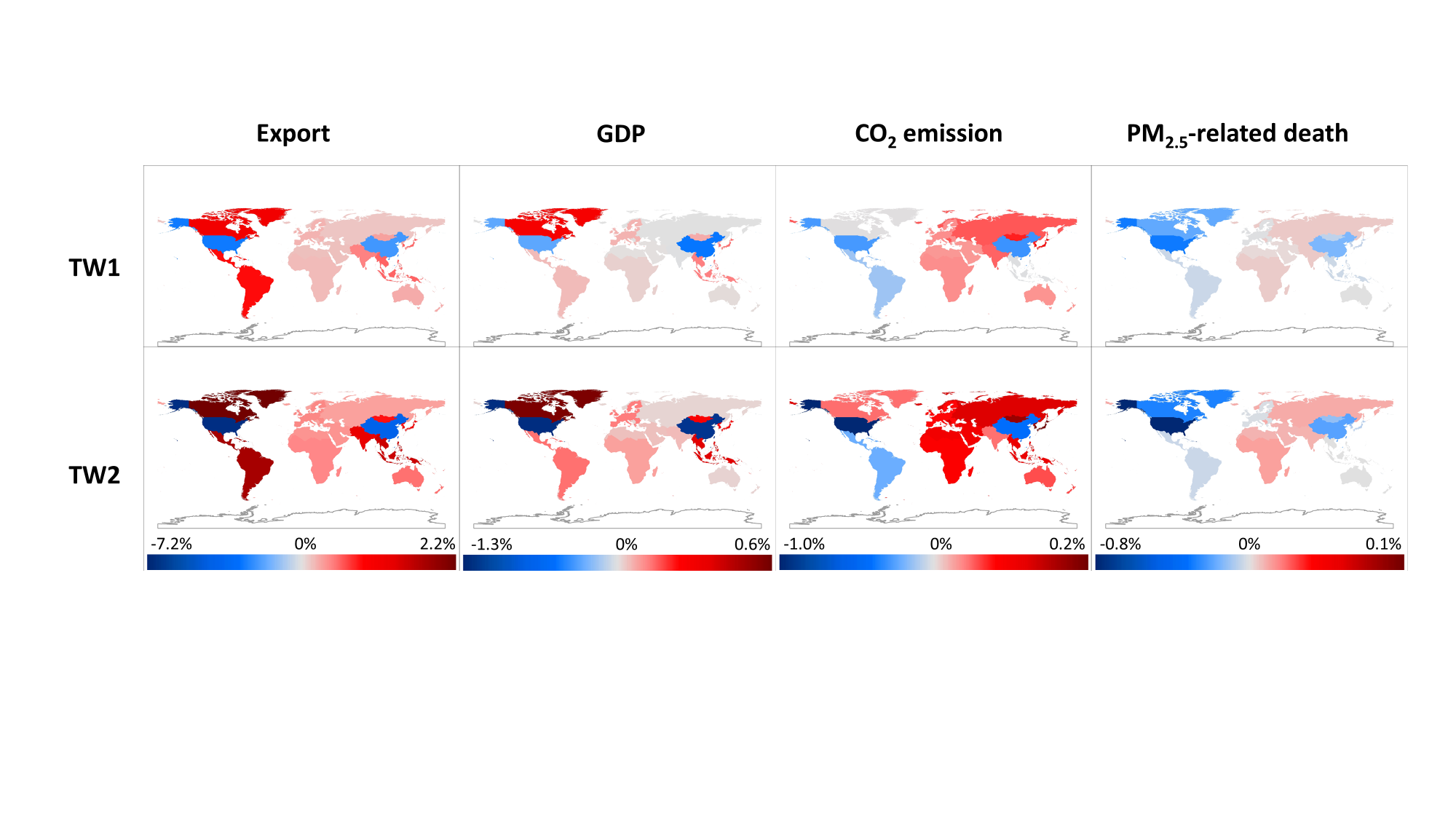


Supplementary Figure 5: Relative changes in regional GDP, CO_2_ emission and PM_2.5_ related premature mortality from actual trade restriction scenario (ATR) to trade war scenario 1 (TW1) and 2 (TW2). Red refers to the increase, while blue refers to the decrease. Note that the results do not include scenario-independent sources.


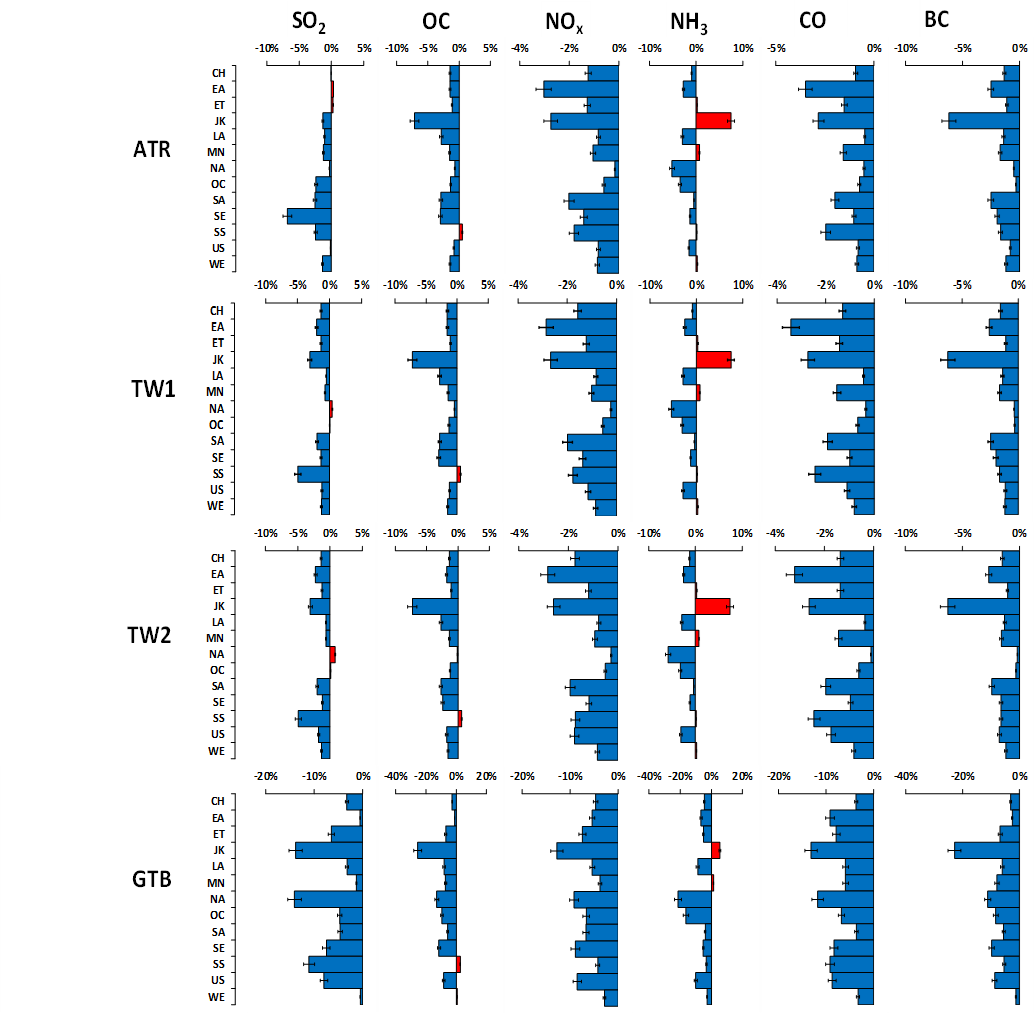
Supplementary Figure 6: Relative changes in regional emissions of six air pollutants emissions from global free trade scenario (GFT) to each alternative scenario. Error bars denote uncertainty ranges (95% CI). Results here only include scenario-dependent sources. Regions include China (CH), rest of East Asia (EA), Economies in Transition (ET), Japan and Korea (JK), Latin America and Caribbean (LA), Middle East and North Africa (MN), rest of North America (NA), Oceania (OC), South Asia (SA), South-East Asia and Pacific (SE), Sub-Saharan Africa (SS), the United States (US), and Western Europe (WE).

Supplementary Figure 7: Relative change in global economic output of each sector from global free trade scenario (GFT) to global trade barrier scenario (GTB). Sectors from top to bottom are ordered by their CO_2_ emission intensities from highest to lowest. The dashed line shows the global average.


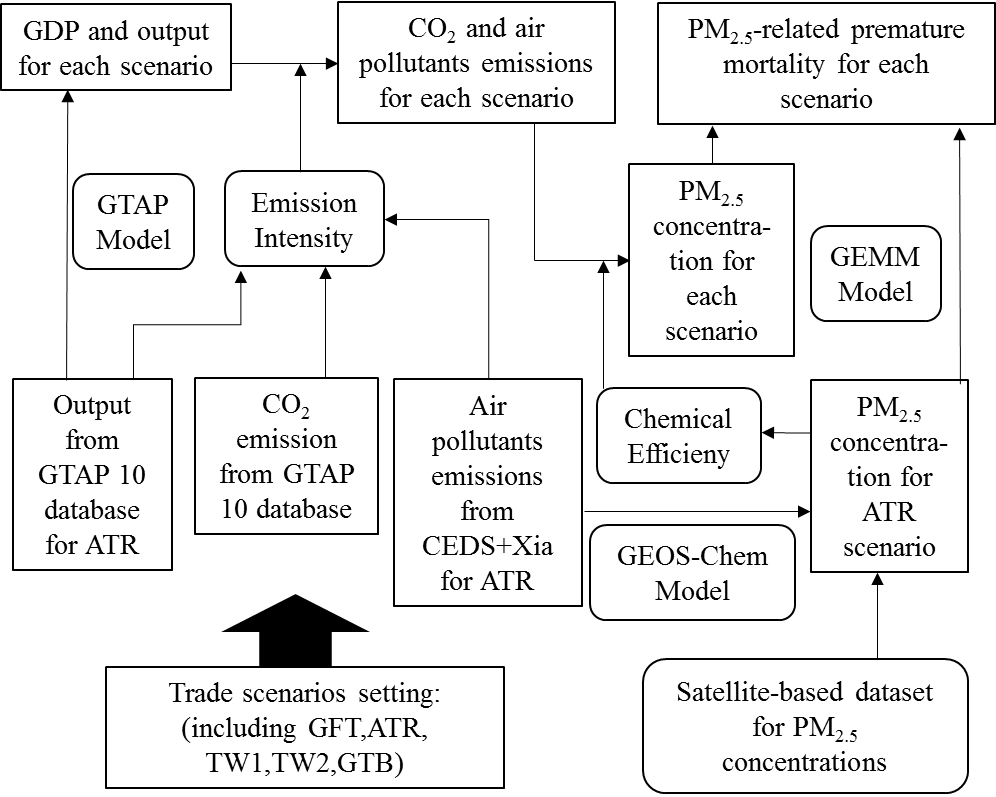


Supplementary Figure 8: Methodology framework of this study.


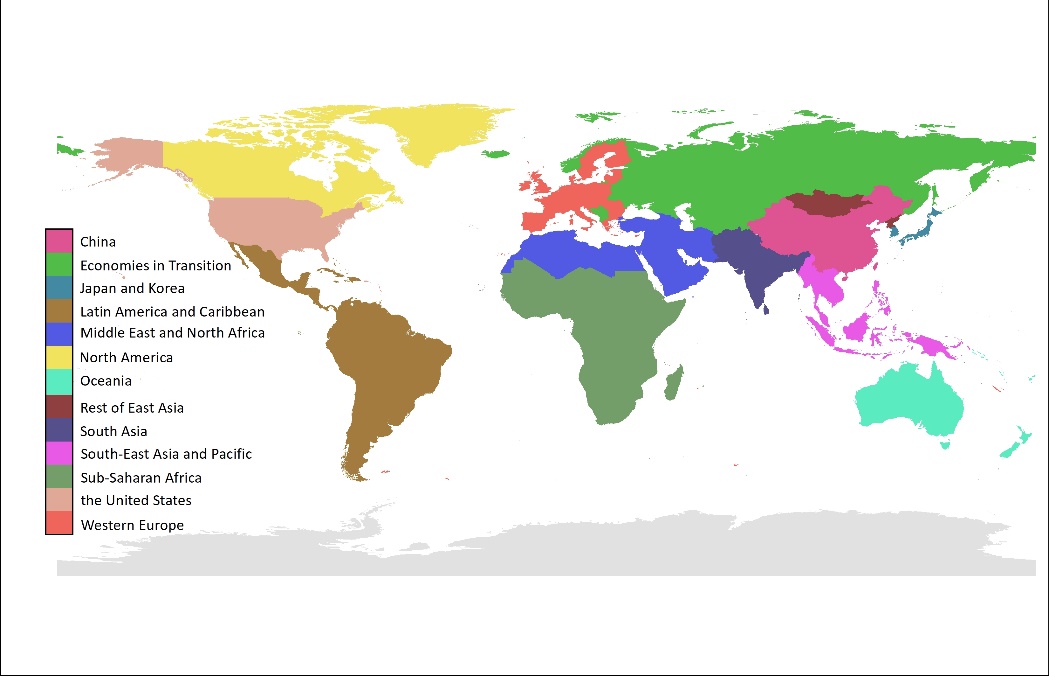


Supplementary Figure 9: The 13 regions used in this study. Regions are aggregated from 141 countries/regions worldwide according to their trade volume, economic volume, and proximity.


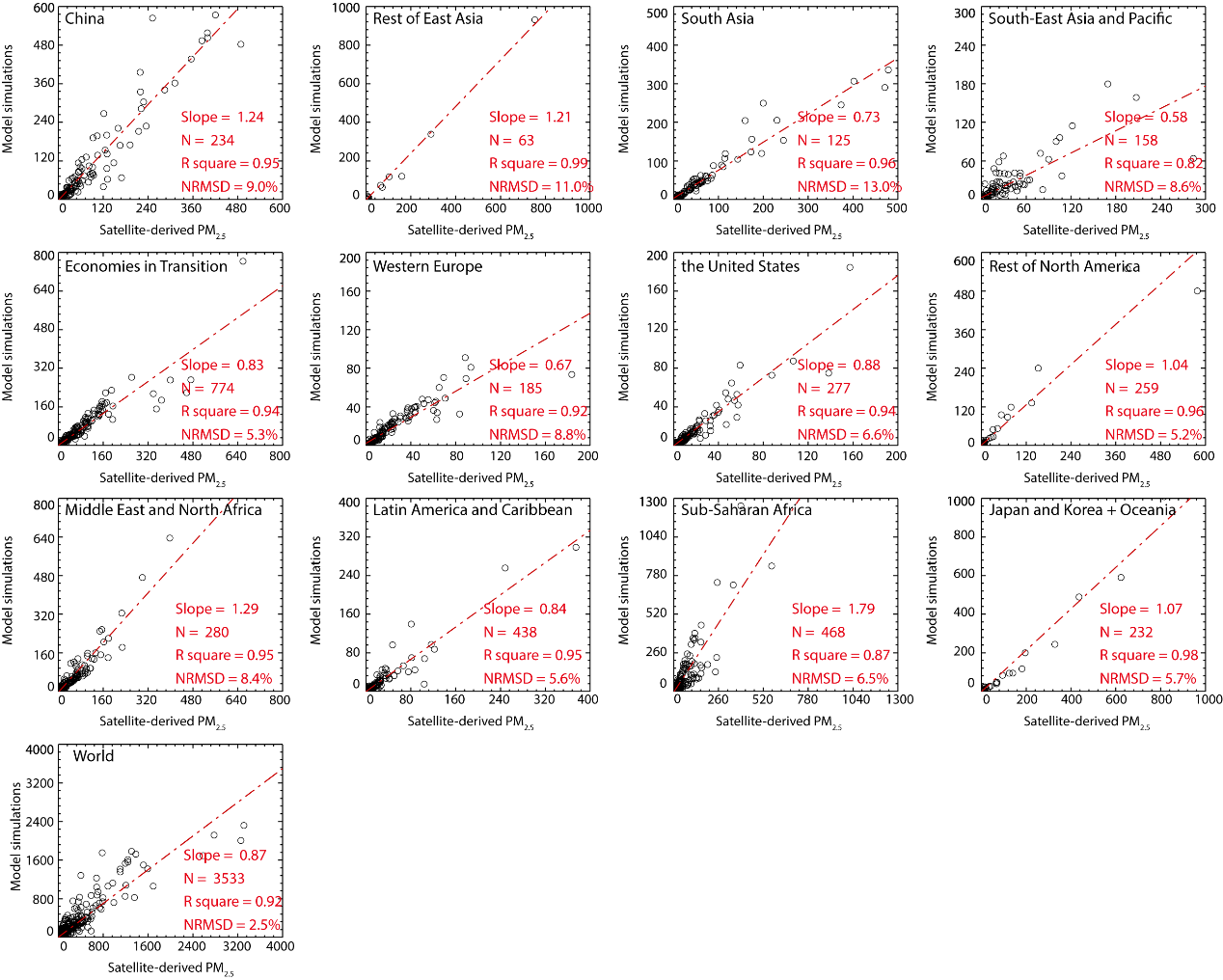


Supplementary Figure 10: Scatter plots for regional PM_2.5_ simulated by the all-emission run of GEOS-Chem and the satellite-derived surface PM_2.5_ data (μg/m^3^). Each data pair represents annual mean population-weighted PM_2.5_ concentration at a given grid cell. Regions dominated by anthropogenic sources are shown in green. Statistical analysis results are also given in each panel. Japan and Korean and Oceania are combined to avoid too few data points.

# Supplementary Tables

**Supplementary Table 1: Comparisons with other studies on the potential relative changes in GDP as a result of the Sino-US trade wars.**

|  | TW1 | | | TW2 | | |
| --- | --- | --- | --- | --- | --- | --- |
|  | China | the US | Global | China | the US | Global |
| This study | -0.64% | -0.39% | -0.14% | -1.22% | -1.26% | -0.36% |
| KPMG | -0.60% | -0.40% | -0.30% | -1% | -0.70% | -0.50% |
| Morgan Stanley | -0.55% | -0.35% | -0.11% |  |  |  |
| Standard & Poor's |  |  |  | -1% | -1.20% |  |

**Supplementary Table 2: The range of imposed tariff of each industry sectors in each scenario. A region on B region means A imposes tariff on goods imported from B.**

|  | **Scenario GFT** | **Scenario TW1** | | **Scenario TW2** | | **Scenario GTB** |
| --- | --- | --- | --- | --- | --- | --- |
|  | **ALL Regions** | **China on the US** | **the US on China** | **China on the US** | **the US on China** | **ALL Regions** |
| Animal products | ALL=0 | ATR+23.88% | ATR+3.81% | ATR+25% | ATR+25% | ATR+25% |
| Beverages and tobacco products | ALL=0 | ATR+19.26% | ATR+4.71% | ATR+25% | ATR+25% | ATR+25% |
| Cereals | ALL=0 | ATR+25% | ATR+10% | ATR+25% | ATR+25% | ATR+25% |
| Chemical; rubber; plastic products | ALL=0 | ATR+7.27% | ATR+7.45% | ATR+25% | ATR+25% | ATR+25% |
| Electricity | ALL=0 | ATR | ATR | ATR+25% | ATR+25% | ATR+25% |
| Fishing | ALL=0 | ATR+19.96% | ATR+8.32% | ATR+25% | ATR+25% | ATR+25% |
| Food products | ALL=0 | ATR+20.72% | ATR+9.27% | ATR+25% | ATR+25% | ATR+25% |
| Forestry | ALL=0 | ATR+9.22% | ATR | ATR+25% | ATR+25% | ATR+25% |
| Leather products | ALL=0 | ATR+1.41% | ATR+10% | ATR+25% | ATR+25% | ATR+25% |
| Machinery and equipment | ALL=0 | ATR+4.18% | ATR+9.71% | ATR+25% | ATR+25% | ATR+25% |
| Metals & products | ALL=0 | ATR+15.49% | ATR+5.93% | ATR+25% | ATR+25% | ATR+25% |
| Minerals & products | ALL=0 | ATR+14.14% | ATR+6.7% | ATR+25% | ATR+25% | ATR+25% |
| Others | ALL=0 | ATR | ATR | ATR+25% | ATR+25% | ATR+25% |
| Paper products; publishing | ALL=0 | ATR+14.43% | ATR+5.54% | ATR+25% | ATR+25% | ATR+25% |
| Road transport | ALL=0 | ATR | ATR | ATR+25% | ATR+25% | ATR+25% |
| Textiles | ALL=0 | ATR+13.88% | ATR+0.82% | ATR+25% | ATR+25% | ATR+25% |
| Transport equipment | ALL=0 | ATR+11.57% | ATR+10.51% | ATR+25% | ATR+25% | ATR+25% |
| Vegetables; fruit; nuts & products | ALL=0 | ATR+24.67% | ATR+6.47% | ATR+25% | ATR+25% | ATR+25% |
| Wearing apparel | ALL=0 | ATR+8.57% | ATR+0.58% | ATR+25% | ATR+25% | ATR+25% |
| Wood products | ALL=0 | ATR+4.87% | ATR+6.32% | ATR+25% | ATR+25% | ATR+25% |

**Supplementary Table 3: Subsector of road transport provided by GAINS. If the flag value is 1, the subsector is considered as private vehicles, otherwise it is considered as commercial vehicles.**

| **sector** | **Flag** |
| --- | --- |
| Passenger cars | 1 |
| Light duty vehicles | 1 |
| Heavy duty vehicles and buses | 0 |
| Mopeds and motorcycles | 1 |

**Supplementary Table 4: Regional mapping from 31 regions to 13 regions.**

| **Region_Name (31)** | **Region_Name (13)** |
| --- | --- |
| China (Mainland) | China |
| Hong Kong, China | China |
| Taiwan | China |
| Central Asia | Economies in Transition |
| Rest of Europe | Economies in Transition |
| Russian Federation | Economies in Transition |
| Japan | Japan and Korea |
| Korea, Republic of | Japan and Korea |
| Brazil | Latin America and Caribbean |
| Mexico | Latin America and Caribbean |
| Rest of Central America | Latin America and Caribbean |
| Rest of South America | Latin America and Caribbean |
| Rest of North Africa | Middle East and North Africa |
| Rest of Western Asia | Middle East and North Africa |
| United Arab Emirates | Middle East and North Africa |
| Australia | Oceania |
| Rest of Oceania | Oceania |
| Rest of East Asia | Rest of East Asia |
| Canada | Rest of North America |
| Rest of North America | Rest of North America |
| India | South Asia |
| Rest of South Asia | South Asia |
| Malaysia | South-East Asia and Pacific |
| Rest of Southeast Asia | South-East Asia and Pacific |
| Singapore | South-East Asia and Pacific |
| Thailand | South-East Asia and Pacific |
| Rest of Sub-Saharan Africa | Sub-Saharan Africa |
| United States of America | the United States |
| European Union | Western Europe |
| Switzerland | Western Europe |
| Rest of the World | / |
