# Supplementary material for: Carbon and health implications of trade restrictions
Source: Nat Commun. 2019 Oct 30;10:4947. doi: 10.1038/s41467-019-12890-3 (PMC6821914; doi:10.1038/s41467-019-12890-3)
Supplement: Supplementary file 3 — Description of Additional Supplementary Files [file 41467_2019_12890_MOESM3_ESM.pdf]

Description of Additional Supplementary Files

File Name: Supplementary Data 1
Description: Tariff of each region and sector from the GTAP 10 database (used in actual trade restriction scenario).

File Name: Supplementary Data 2
Description: PM2.5 related mortality results based on the GEMM NCD+LRI, GEMM 5COD and IER method. PM2.5 considered here only include scenario-dependent SIOA, BC and POA together.

File Name: Supplementary Data 3
Description: Regional mapping and sectoral mapping from GTAP to this study.

File Name: Supplementary Data 4
Description: Sectoral mapping from Xia et al. inventory to CEDS.

File Name: Supplementary Data 5
Description: The fractional contributions of emissions from private vehicles to the total emissions from the transportation sector.

File Name: Supplementary Data 6
Description: Regional mapping and sectoral mapping from CEDS to GTAP.
